# Obesity and diabetes cause cognitive dysfunction in the absence of accelerated β-amyloid deposition in a novel murine model of mixed or vascular dementia

**DOI:** 10.1186/2051-5960-2-64

**Published:** 2014-06-10

**Authors:** Dana M Niedowicz, Valerie L Reeves, Thomas L Platt, Katharina Kohler, Tina L Beckett, David K Powell, Tiffany L Lee, Travis R Sexton, Eun Suk Song, Lawrence D Brewer, Caitlin S Latimer, Susan D Kraner, Kara L Larson, Sabire Ozcan, Christopher M Norris, Louis B Hersh, Nada M Porter, Donna M Wilcock, Michael Paul Murphy

**Affiliations:** Sanders Brown Center on Aging, University of Kentucky, 800 S. Limestone, Sanders Brown 211, Lexington, KY 40536-0230 USA; Department of Molecular and Cellular Biochemistry, University of Kentucky, Lexington, USA; Department of Molecular and Biomedical Pharmacology, University of Kentucky, Lexington, USA; Magnetic Resonance Imaging and Spectroscopy Center, University of Kentucky, Lexington, USA

**Keywords:** Dementia, Diabetes, Obesity, Stroke, Alzheimer’s disease

## Abstract

Mid-life obesity and type 2 diabetes mellitus (T2DM) confer a modest, increased risk for Alzheimer’s disease (AD), though the underlying mechanisms are unknown. We have created a novel mouse model that recapitulates features of T2DM and AD by crossing morbidly obese and diabetic *db/db* mice with *APP*^*ΔNL/ΔNL*^*x PS1*^*P264L/P264L*^ knock-in mice. These mice (*db/AD*) retain many features of the parental lines (*e.g.* extreme obesity, diabetes, and parenchymal deposition of β-amyloid (Aβ)). The combination of the two diseases led to additional pathologies-perhaps most striking of which was the presence of severe cerebrovascular pathology, including aneurysms and small strokes. Cortical Aβ deposition was not significantly increased in the diabetic mice, though overall expression of presenilin was elevated. Surprisingly, Aβ was not deposited in the vasculature or removed to the plasma, and there was no stimulation of activity or expression of major Aβ-clearing enzymes (neprilysin, insulin degrading enzyme, or endothelin-converting enzyme). The *db/AD* mice displayed marked cognitive impairment in the Morris Water Maze, compared to either *db/db* or *APP*^*ΔNL*^*x PS1*^*P264L*^ mice. We conclude that the diabetes and/or obesity in these mice leads to a destabilization of the vasculature, leading to strokes and that this, in turn, leads to a profound cognitive impairment and that this is unlikely to be directly dependent on Aβ deposition. This model of mixed or vascular dementia provides an exciting new avenue of research into the mechanisms underlying the obesity-related risk for age-related dementia, and will provide a useful tool for the future development of therapeutics.

## Introduction

Alzheimer’s disease (AD) is a neurodegenerative disease affecting the elderly. There are two major neuropathologies associated with AD: extracellular plaques containing β-amyloid (Aβ) and intracellular neurofibrillary tangles composed of the microtubule-associated protein tau. The combined insults of Aβ and tau accumulation are thought to promote the progressive synaptic failure and neuronal loss, leading to memory loss and cognitive impairment [1–4]. While familial forms of AD exist, sporadic AD is far more common. Though the two forms of AD ultimately reflect similar pathologies, the underlying causes vary. Familial AD is linked to specific mutations in amyloid precursor protein (APP) or presenilin (PS1 or PS2), leading to accumulation of toxic β-amyloid species in the brain by mid-life. Sporadic AD manifests later in life, and the triggers are less clear and likely complex. Though there are genetic components associated with sporadic AD, environmental factors, such as lifestyle (*e.g.* diet and exercise), are also likely to impact disease onset and progression.

Obesity is a major worldwide public health problem, and is associated with the metabolic disorder type 2 diabetes mellitus (T2DM). Diabetes is associated with cognitive decline in both rodents and humans [5–7]. Due to improved treatments, T2DM patients are living longer, putting them at increased risk for age-related complications. Although simply living to an older age increases the risk of Alzheimer’s disease, there is a well-known (albeit poorly understood) link between obesity, T2DM and dementia [8]. The form of dementia afflicting these individuals combines elements of vascular pathology, small strokes and AD-related neuropathology. In fact, the amount of AD pathology is essentially unchanged in cases with a history of T2DM, while cerebrovascular pathology increases [9, 10]. Vascular dementia, or even cerebrovascular dysfunction as a general AD comorbidity, is a poorly understood condition with no viable treatment options. This is due to cerebrovascular dysfunction being understudied as a major cause of dementia and the lack of useful model systems in which to develop therapies or to study the disease process.

In this paper, we describe the creation of a novel mouse model combining the key features of obesity, diabetes, and AD. We crossed the obese and diabetic *db/db* mouse [11–13] with the APP^ΔNL/ΔNL^ × PS1^P264L/P264L^ knock-in model of AD [14, 15]. The resulting mice (which we have called *db/AD*) are morbidly obese, glucose intolerant, insulin resistant, and display parenchymal amyloid plaques, similar to the parental lines. In addition, although these mice had profound cognitive impairment and marked cerebrovascular abnormalities, this does not appear to be driven by Aβ deposition. The db/AD mice will be a useful tool with which to study the intersection of T2DM and dementia.

## Materials and methods

### Mouse breeding

In order to create a diabetic AD mouse model, we crossed the obese, diabetic *Lepr*^*db/db*^ (*db/db*) mice [11–13] with the *APP*^*ΔNL/ΔNL*^*/PS1*^*P264L/P264L*^ (APP/PS1) knock-in model of AD [14, 15]. Because the homozygous *db/db* mice are infertile, heterozygous (*Lepr*^*db/+*^) mice on a C57Bl/6 J background (Jackson Labs; Bar Harbor, ME) were bred with *APP/PS1* mice on a CD-1/129 background (obtained from the breeding colony at the University of Kentucky). The resulting F1 mice heterozygous for all three alleles were then intercrossed to generate wild-type, heterozygous, and homozygous *db* mice that were either wild-type or homozygous for the AD knock-in genes. For most of the data presented here, we focused on four main genotypes: wild-type (WT; *Lepr*^*+/+*^ × *APP*^*+/+*^*/PS1*^*+/+*^), db (*Lepr*^*db/db*^ × *APP*^*+/+*^*/PS1*^*+/+*^), AD (*Lepr*^*+/+*^ × *APP*^*ΔNL/ΔNL*^*/PS1*^*P264L/P264L*^), and db/AD (*Lepr*^*db/db*^*x APP*^*ΔNL/ΔNL*^*/PS1*^*P264L/P264L*^). Some analyses included *Lepr*^*db/+*^*APP*^*+/+*^*/PS1*^*+/+*^ and *Lepr*^*db/+*^ × *APP*^*ΔNL/ΔNL*^*/PS1*^*P264L/P264L*^ mice (noted where appropriate). Mice were housed under a 12 hour light–dark cycle and fed standard rodent chow *ad libitum*. Mice were euthanized by CO_2_ asphyxiation, followed by decapitation. All animal work was conducted with prior University of Kentucky (UK) IACUC approval, and was performed in accordance with USDA and PHS guidelines.

### Genotyping

Tail snips were collected prior to weaning. For some of the *db* and *APP* genotyping, tail snips were sent to Transnetyx (Cordova, TN) for purification and analysis. For those analyzed in our lab, as well as *PS1* genotyping, genomic DNA was isolated and purified from tail snips using the Promega Wizard Genomic DNA kit (Promega; Madison, WI). *db* genotyping was performed using a single nucleotide polymorphism Taqman® genotyping kit (Applied Biosystems Life Technologies; Grand Island, NY). *APP* and *PS1* genotyping were performed by PCR as described previously [16] using GoTaq® Flexi DNA Polymerase (Promega).

### Mouse groups

The mice used for this study were broadly divided by age and will be referred to as young (1–4 months old: 3.0 ± 0.8 months), middle-aged (5–9 months old: 7.2 ± 1.6 months), and older (10–14 months old: 12.2 ± 1.0 months) based on the predicted lifespan of the *db/AD* mice (~15-16 months).

### Glucose and insulin tolerance tests

Mice were fasted 3–6 hours prior to the start of the glucose tolerance test (GTT) or insulin tolerance test (ITT). All glucose measurements were obtained via tail bleed using a Bayer Breeze 2 glucometer and test strips (Bayer; Tarrytown, NY). For the GTT, a baseline measurement was obtained after which the GTT was initiated by intraperitoneal injection of dextrose (2 mg/g: Hospira; Lake Forrest, IL). Subsequent measurements were recorded at 15, 30, 60, and 120 minutes post-injection. For the ITT, a baseline glucose measurement was taken, after which insulin (0.75 U//kg: Eli Lilly; Indianapolis, IN) was injected intraperitoneally. Subsequent measurements were recorded at 15, 30, 60, and 120 minutes post-injection. Any glucometer reading of “HI” was set to 700 mg/dL for data analysis.

### Blood pressure measurements

Blood pressure (BP) was measured using a Kent CODA 8 BP machine (Kent Scientific; Torrington, CT). Animals were allowed to acclimate to the tail blood pressure cuff for five minutes on a warming platform before recording BP measures. The BP measures consisted of 20 cycles of diastolic/systolic measures, with a 20 second rest period between cycles. After finishing the data collection, the mice were immediately released back into their home cages. The rodent restraints, cuffs, and warming platform were cleaned between animals; female animals were always run after male animals to avoid any possible irritation of the males. BP measures were performed at the same time each day to account for the possible influence of circadian rhythms.

### Plasma measurements

Blood was collected upon decapitation in the presence of EDTA, centrifuged (1500 × *g*, 10 min.), and the plasma collected. Plasma leptin was measured by a commercially-available, species-specific ELISA (EMD Millipore; Billerica, MA), according to package instructions.

### Immunoassays

Frozen brain tissue was serially extracted in either PBS or HEPES (20 mM HEPES, 2 mM EDTA, 2 mM EGTA, 0.32 M sucrose) followed by 2% SDS, and 70% formic acid as previously described [17, 18]. Buffers were supplemented with protease inhibitor cocktail (Amresco; Solon, OH) and phosphatase inhibitor cocktail (EMD Millipore). The tissue was homogenized using an AHS200 PowerMax (VWR; Radnor, PA) homogenizer, the insoluble material was removed by centrifugation (PBS/HEPES/SDS: 20,800 × *g*, 30 minutes; formic acid; 20,800 × *g*, 60 min) and the supernatants frozen until use. Human-specific Aβ was measured by two-site sandwich ELISA as previously described [17]. Oligomeric Aβ (mouse and human) was measured by single-site sandwich ELISA as previously described [19, 20]. Briefly, 384-well plates (Immulon 4HBX: Thermo Scientific; Waltham, MA) were coated with either 0.5 μg Ab42.5 (Aβ_total_ and Aβ_1–40_), Ab2.1.3 (Aβ_1–42_), or 4G8 (oligomers: Covance, Princeton, NJ)/well and blocked with Synblock (Serotec; Raleigh, NC) for two hours. PBS and SDS extracts were diluted in AC buffer (0.2 M sodium phosphate (pH7), 0.4 M NaCl, 2 mM EDTA, 0.4% Block Ace (Serotec), 0.4% BSA, 0.05% CHAPS, 0.05% NaN_3_) for analysis. Formic acid extracts were first neutralized with TP buffer (1 M Tris base, 0.5 M sodium phosphate: 20-fold dilution), then further diluted with AC buffer for analysis. Similarly, plasma was diluted in AC buffer for analysis. A standard curve was prepared from recombinant human Aβ_1–42_, Aβ_1–40_, or oligomeric Aβ diluted in AC buffer. Standards and samples were measured at least in duplicate. After incubation with the samples and standards, Aβ was detected with either biotinylated-4G8 (Aβ_total_, Aβ_1–42_, and oligomers: Covance) or biotinylated-13.1.1 (Aβ_1–40_), followed by incubation with 0.1 μg/mL NeutrAvidin-HRP (Pierce Technologies; Rockford, IL). The plate was developed with 3′,3′,5′,5′-tetramethylbenzidine (Kirkeguard and Perry Laboratories; Gaithersburg, MD) and the reaction stopped with 6% *o*-phosphoric acid. The absorbance at 450 nm was measured with a BioTek (Winooski, VT) multiwell plate reader.

Protein levels of PS1, BACE1, BACE2, phosphorylated and total tau, endothelin-converting enzyme 1 (ECE1), and PSD95 were determined by Western or spot blot, using protein-specific antibodies (PS1 (EMD Millipore), BACE1 (Epitomics; Burlingame, CA), BACE2 (Abcam; Cambridge, MA), pTau (AT8: Sigma-Aldrich; St. Louis, MO), total tau (HT7: Pierce: [21, 22]), ECE1 (Acris Antibodies; San Diego, CA), PSD95 (D27E11; Cell Signaling; Danvers, MA)). Immunoreactive bands for PS1, BACE1, BACE2, tau, and ECE1 were visualized with Super Signal West Dura chemiluminescence HRP substrate (Pierce) after incubation with HRP-conjugated secondary antibodies and exposed to film. Densitometric analyses were performed using Image J software. Expression was standardized to β-actin (Sigma-Aldrich) or GAPDH (Abcam) expression in the same lane or spot, respectively. PSD95 and its GAPDH loading control (Abcam) were visualized with fluorescently-labeled secondary antibodies (LI-COR; Lincoln, NE) using an Odyssey Infrared Imager (LI-COR) for quantitation and analysis.

### qPCR

Tissue was homogenized in Trizol™ (Invitrogen; Grand Island, NY) in order to isolate RNA, followed by phenol/chloroform extraction. When needed, RNA was further purified by RNeasy columns (Qiagen; Valencia, CA). Expression of ECE1 and ECE2 were determined by two-step qRT-PCR, using iScript (BioRad; Hercules, CA) reverse transcription, followed by qPCR with PerfeCTa FastMix™ (Quanta BioSciences; Gaithersburg, MD). The geometric mean of the C_T_ values for RPL30, cyclophilin, and RNA polymerase IIJ was used as an internal control to calculate and compare relative expression (2^-ΔΔC^_T_). Gene specific primer sets were obtained from IDT (Coralville, IA).

### Neprilysin and insulin degrading enzyme activity

Neprilysin (NEP) activity was measured as described [23]. Briefly, hemibrains were homogenized in ice-cold Tris buffer (50 mM Tris–HCl and 150 mM NaCl, pH 7.2; 100 mg/mL) supplemented with 1 mM PMSF (Sigma-Aldrich), and 10 μM E-64 (RPI; Mt. Prospect, IL). The homogenate was centrifuged (1000 × *g*, 20 min., 4°C), followed by a high-speed centrifugation of the supernatant (100,000 × *g*, 1 hour, 4°C). The supernatant was removed, and the pellet resuspended in Tris buffer for the enzyme assay. NEP activity was measured using glutaryl-Ala-Ala-Phe-4-methoxy-2-naphthylamide (Sigma-Aldrich) as a substrate. Reactions were initiated with the addition of the membrane fraction, then fluorescent product formation was monitored (340 nm excitation, 425 nm emission, 37°C). Phosphoramidon (50 μM) and thiorphan (10 μM) were used to inhibit NEP activity and determine background fluorescence for each sample.

Insulin degrading enzyme (IDE) activity was measured using a commercially-available kit (EMD Millipore) according to manufacturer’s instructions. Briefly, hemibrains were homogenized in Tris buffer (100 mg/mL) supplemented with PMSF and E-64, centrifuged (20,800 × *g*, 30 min., 4°C), and the supernatant used for the activity assay. Samples were compared against rat IDE. Fluorescence was measured at an excitation wavelength of 320 nm and an emission wavelength of 405 nm.

### MRI

T2*-MRI was performed using a horizontal bore Bruker Clinscan (7.0 T, 30 cm, 300 MHz: Billerica, MA) imager equipped with a triple-axis gradient (630 mT/m and 6300 T/m/s) and a helium-cooled 14 K quadrature head cryo-coil, cooled to 20°K. T2*-weighted images were acquired with a 2D GRE sequence with at 34 μm × 34 μm × 400 μm resolution, 15 mm FOV, 25 degree flip angle, 10 averages, TR 165 ms, and TE 15.3 ms. Mice were imaged under constant isofluorane anesthesia and their body temperature and respiration were continuously monitored. At least ten equally-spaced images were taken of each mouse brain. Asymmetrically-occurring dark spots on the images were considered indicative of vascular events (confirmed histologically, see below), whereas symmetrically-occurring dark areas were considered to be blood vessels and were excluded.

### Vascular corrosion casting

Vascular corrosion casting was performed as described [24]. Briefly, mice were anesthetized using pentobarbital (100 mg/kg), followed by transcardial perfusion with heparinized saline (0.9%). Following a brief perfusion with *para*-formaldehyde (4%), the brains were perfused with the polyurethane resin Pu4ii (4 mL/min: VasQtec; Switzerland). After allowing the resin to cure for at least two days, the brains were incubated in KOH (7.5%, 50°C, 48 h), followed by formic acid (5%, 50°C, 24 h). The tissue was subsequently frozen, then lyophilized to macerate the soft tissue. Finally, the casted brains were sputter-coated in palladium and viewed by scanning electron microscopy (Hitachi S-4300: Schaumburg, IL), using the middle cerebral artery as a landmark. Endothelial cell density was determined by endothelial cell nuclear imprints measured directly using Image J software. Aneurysm pathology was assessed on a 4 point scale based on clear data break points (0 = none; 1 = 1 possible; 2 = 1–3 definite; 3 = 4+ definite. Vascular density was determined by rank order of representative images using three blinded, independent reviewers. Images were scored from 1 (most dense) – 26 (least dense), and the ranks from the three reviewers averaged.

### Histology

Tissue was harvested and fixed in PBS-buffered 10% formalin for at least 24 hrs. For Aβ immunohistochemistry, hemibrains were embedded in a matrix and sectioned (30 μm) by NeuroScience Associates (Knoxville, TN). For Prussian blue staining, hemibrains were embedded in paraffin and sectioned to 8 μm using a microtome. For free-floating sections, the hemibrains were incubated in sucrose (10%, 20%, 30% sequentially for 24 hours each) for cryoprotection, then sectioned on a sliding, freezing microtome to 25 μm.

Perl’s Prussian blue staining of hemosiderin was performed as described [25]. Immunohistochemistry detecting Aβ was performed using antibody 4G8 (Covance) as described [19]. Some Aβ immunohistochemistry was performed by NeuroScience Associates. Densitometry was performed on these sections using Image J software. Vascular Aβ was visualized by three different methods: 1) Congo red (0.2% in NaCl-saturated 80% ethanol), 2) Thioflavin S (1%: Sigma-Aldrich), and 3) resorufin (Sigma-Aldrich: [26]). Cerebral blood vessels were imaged in free-floating sections using a mouse anti-α-actin antibody (A5228: Sigma-Aldrich), followed by quantitation with Image J software. Triple labeling of free-floating sections was performed with the fluorescent Aβ-specific Amylo-Glo stain (Biosensis; Thebarton, Australia), rabbit anti-collagen IV (ab6586: Abcam), and rabbit anti-glial fibrillary acidic protein (G9269: Sigma-Aldrich).

### Behavioral testing

Testing was performed by the UK Rodent Behavioral Core (http://www.rodentbehaviorcore.uky.edu/default.aspx/0_UK_Rodent_Behavior_Core). Mice were tested using the Morris Water Maze paradigm. The maze consisted of a circular pool (134.5 cm diameter) filled with 25°C water. A circular platform (11 cm diameter) was placed in the northeast quadrant 1 cm below the surface of the water so that it was not visible. Nontoxic tempura paint was used to create opaque water, thus obscuring the platform. The pool was placed behind dark curtains holding external maze cues. The cues were rotated each day. There were five consecutive training/acquisition days. On each-training day the animals swam four trials (rotating initial placement each time), lasting one minute each, with a five minute interval between trials. After a 30 minute rest upon the conclusion of training on the fifth day, we performed a probe trial where the platform was removed from the pool. The animal’s location in the pool was recorded for one minute and used to calculate the time spent in the target quadrant and the number of times crossing the platform area. After the completion of training, mice were tested for visual acuity during which the external cues were provided along with a visibly-raised platform. The mice were tested for visual acuity in four trials during one day. Water Maze data (e.g. swim speed, distance, latency to platform. etc.) were collected and analyzed using EthoVision XT software (Noldus Information Technology; Leesburg, VA).

### Passive immunization

A small number of db/AD mice (N = 7; 9–12 month old; 3 M/4 F) were injected intraperitoneally with Ab42.5 (300 μg in sterile saline) every two weeks for two months. Mice were imaged by T2* MRI prior to starting the treatment (baseline) and prior to death (endpoint). The majority of the brains (N = 6; 2 M/4 F) were extracted in RIPA buffer (50 mM Tris–HCl, 150 mM NaCl, 1% Triton X-100, 0.5% deoxycholate, 0.1% SDS; pH = 8.0) with protease inhibitor cocktail (Amresco) for Aβ_total_ ELISA measurement as described above. Brains from untreated, age-matched db/AD mice (N = 6; 2 M / 4 F) were also extracted in RIPA and used as controls. Endpoint MRI scans were compared against untreated, age-matched (11–14 months old) db/AD mice.

### Statistics

Weight data were analyzed by student’s t-test at each age using Microsoft Excel, and the probability adjusted using the Holm-Bonferroni method [27]. All other data were analyzed with SPSS (Hewlett Packard; Palo Alto, CA) using the general linear model (GLM) module for ANOVA with the independent variables gender, db genotype, and AD genotype (for an explanation of this model, see http://pic.dhe.ibm.com/infocenter/spssstat/v21r0m0/index.jsp?topic=%2Fcom.ibm.spss.statistics.help%2Fidh_glm_multivariate.htm). Post-hoc multiple comparisons were conducted using Tukey’s test, Dunnett’s test, or similar. Chi-square analyses were performed on the visual acuity measurements for the Morris Water Maze. We performed correlation analyses using either Pearson’s *r* or Spearman’s ρ (parametric and nonparametric values, respectively), and adjusted probability using the Holm-Bonferroni method. For nonparametric comparisons, we used a Kruskal-Wallis ANOVA, or Mann–Whitney U test, where appropriate. For most presented statistics, we note an overall effect of genotype (*db* or *AD*) across the data set. In some cases, we also present a direct comparison between two different genotypes.

## Results

### Creation and characterization of the db/AD mouse model

**Figure 1:**
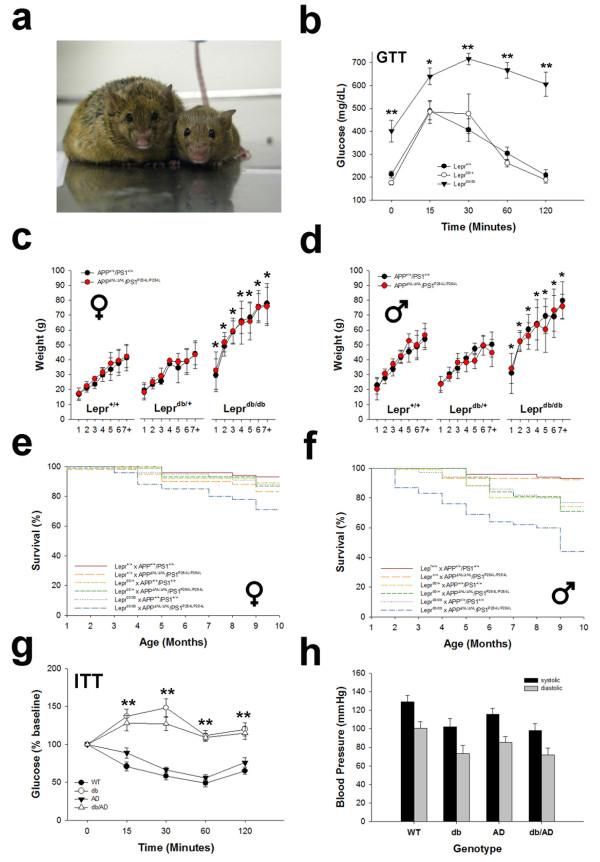
***db/AD*** **characteristics.** The diabetic *Lepr* ^*db/db*^ mouse was crossed to the *APP/PS1* knock-in model of AD to create the *db/AD* line. **(a)** Mice homozygous for the db gene were obese (female, 9 months old; *left: Lepr* ^*db/db*^ mouse; *right: Lepr* ^*db/+*^ *mouse*). **(b)** *Lepr* ^*db/db*^ mice showed an impaired response to glucose (F[2,30] = 38.2, p ≤ 0.0001 for the *db* genotype overall, n = 13 *Lepr* ^*+/+*^, n = 5 *Lepr* ^*db/+*^, n = 18 *Lepr* ^*db/db*^; ** = p ≤ 0.01, * = p ≤ 0.05, Tukey’s LSD). The *AD* genotype did not affect the GTT (*not shown*). **(c - d)** *Lepr* ^*db/db*^ mice showed substantial weight gain from an early age. Weight was relatively stable after ~7 months, and there was no effect of the AD genotype (* = p ≤ 0.01, t-test; Holm-Bonferroni [27] correction; n = ~14 mice / genotype). Female **(e)** and male **(f)** *Lepr* ^*db/db*^ *x APP* ^*ΔNL/ΔNL*^ */PS1* ^*p264L/P264L*^ mice had reduced survivability compared with other genotypes, though the males had a particularly high attrition rate*.* (N = 367 F/359 M: *Lepr* ^*db/db*^ *x APP* ^*ΔNL/ΔNL*^ */PS1* ^*p264L/P264L*^, n = 56 F/53 M; *Lepr* ^*db/db*^ *x APP* ^*+/+*^ */ PS1* ^*+/+*^ *,* n = 73 F/60 M; *Lepr* ^*+/+*^ x *APP* ^*ΔNL/ΔNL*^ */PS1* ^*p264L/P264L*^, n = 54 F/44 M; *Lepr* ^*+/+*^ x *APP* ^*+/+*^ */ PS1* ^*+/+*^, n = 56 F/61 M; *Lepr* ^*db/+*^ × *APP* ^*ΔNL/ΔNL*^ */PS1* ^*P264L/P264L*^, n = 69 F/61 M; *Lepr* ^*db/+*^ × *APP* ^*+/+*^ */ PS1* ^*+/+*^, n = 59 F/80 M) In subsequent experiments, we focused on the four main genotypes. For simplicity we have named them *WT*, *db*, *AD*, and *db/AD*. **(g)** *Lepr* ^*db/db*^ (*db* and *db/AD*) mice were insulin resistant (F[15,177] = 4.64, p ≤ 0.0001 for the *db* genotype overall; *WT*, n = 14; *db*, n = 13; *AD*, n = 20; *db/AD*, n = 18; ~6 months of age; * = p ≤ 0.05, ** = p ≤ 0.01; Dunnett’s test, relative to *Lepr* ^*+/+*^ (*WT* and *AD*)). **(h)** Blood pressure was not different, although there was a modest tendency (F[2,15] = 3.24, p ≤ 0.1) for it to be slightly *lower* overall in *Lepr* ^*db/db*^ mice regardless of the AD genotype, at least at this age (n = 5–7 mice / genotype, ~7-8 months of age).

In order to explore the mechanisms underlying the increased risk for AD in T2DM patients, we created a unique mouse model that recapitulates features of both diseases. The obese, leptin-resistant, and diabetic *db/db (Lepr*^*db/db*^) mouse was crossed with the *APP*^*ΔNL/ΔNL*^*x PS1*^*P264L/P264L*^ knock-in model of AD. Since *APP* and *PS1* are under the control of their endogenous promoters, expression follows normal murine levels and patterns. The resulting mice homozygous for the *Lepr*^*db*^ mutation were morbidly obese (Figure 1a). In addition, young *Lepr*^*db/db*^ mice displayed elevated fasting glucose and impaired glucose tolerance (Figure 1b: F[2, 30] = 38.2, p < 0.0001). On average, *Lepr*^*db/db*^ mice were 2-fold heavier than *Lepr*^*+/+*^ and *Lepr*^*db/+*^ mice (Figure 1c-d: p ≤ 0.001 for all ages). The AD genotype had no effect on weight (p ≥ 0.3 for all ages). Consistent with their increased weight, older *Lepr*^*db/db*^ mice had significantly more plasma leptin (168 ± 15 ng/mL; n = 9 F/17 M) than *Lepr*^*+/+*^ (54 ± 15 ng/mL; n = 6 F/20 M) or *Lepr*^*db/+*^ (49 ± 15; n = 11 F/15 M; p ≤ 0.0001: *not shown*) animals. The AD genotype had no effect on circulating leptin (*APP*^*+/+*^ × *PS1*^*+/+*^ 81 ± 12 ng/mL, n = 12 F/26 M vs. *APP*^*ΔNL/ΔNL*^*× PS1*^*P264L/P264L*^ 99 ± 12 ng/mL, n = 15 F/23 M; p = 0.3). The *Lepr*^*db/db*^*× APP*^*ΔNL/ΔNL*^*/PS1*^*p264L/P264L*^ mice exhibited decreased survival compared with all other genotypes, particularly in the males, which had a ~50% attrition rate by 10 months (Figure 1e-f). Since there is little apparent difference in the tested metabolic parameters between *Lepr*^*+/+*^ and *Lepr*^*db/+*^ mice, we chose to focus on the *Lepr*^*+/+*^ and *Lepr*^*db/db*^ genotypes for subsequent experiments. For simplicity, we will refer to the four main genotypes as WT (*Lepr*^*+/+*^ × *APP*^*+/+*^*/PS1*^*+/+*^), db (*Lepr*^*db/db*^*× APP*^*+/+*^*/PS1*^*+/+*^), AD (*Lepr*^*+/+*^ × *APP*^*ΔNL/ΔNL*^*/PS1*^*P264L/P264L*^), and db/AD (.*Lepr*^*db/db*^*x APP*^*ΔNL/ΔNL*^*/PS1*^*P264L/P264L*^). Middle-aged *db* and *db/AD* mice also had impaired insulin sensitivity (Figure 1g: F[15,177] = 4.64, p ≤ 0.0001). Collectively, these data indicated that the *db* homozygous mice had metabolic dysfunction. Blood pressure was not different in middle-aged diabetic mice compared with nondiabetics, although there was a modest tendency (Figure 1h: p ≤ 0.1) for it to be slightly *lower* overall.

### β-amyloid

**Figure 2:**
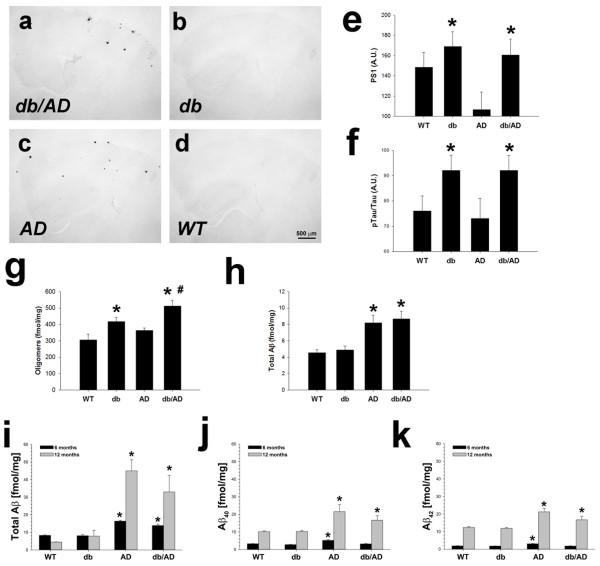
**Amyloid pathology in** ***db/AD*** **mice. (a - d)** Aβ deposition (4G8 IHC) in mouse neocortex (~8 months old: *magnification:* 4×). *db/AD* **(a)** and AD **(c)** mice displayed amyloid-containing plaques, while *db* **(b)** and *WT* **(d)** mice did not. The *db* genotype significantly upregulated both PS1 expression (p < 0.002 for *db* overall: **e)** and tau phosphorylation (p < 0.003 standardized to total tau: **f)** in the brain (middle-aged: n = 10 F/12 M *db* wild-type; 13 F/11 M *db* homozygotes; 22 F/28 M heterozygotes). The results are similar for phosphor-tau when it is not standardized to total tau (*not shown*) **(g)** Aβ oligomers were significantly increased in diabetic (*db* and *db/AD)* mice (p < 0.001 for *db* overall, *WT*, n = 8; *db*, n = 8; *AD*, n = 8; *db/AD*, n = 7) compared to non-diabetic mice. Additionally, oligomers were significantly higher in *db/AD* compared to *AD* mice (^**# =**^ p < 0.0005). **(h)** Young (3 month old) *db/AD* mice did not have significantly more Aβ than *AD* mice (n = 5 F/1 M *WT*; 5 F/1 M *db*; 3 F/3 M *AD*; 3 F/3 M *db/AD*: p < 0.29). **(i)** Total Aβ (Ab42.5/4G8), **(j)** Aβ_1–40_ (Ab42.5 / 13.1.1), and **(k)** Aβ_1–42_ (Ab2.1.3 / 4G8) all increased with age (p ≤ 0.0001); the *db* genotype had no overall effect, although there was a modest reduction in Aβ_1–42_ (p ≤ 0.01). * = p ≤ 0.05, relative to *db* WT. N = 77 [genotype, age (6–7 mo./12 mo.): *WT*, n = 13/8; *db*, n = 13/8; *AD*, n = 9 / 8; *db/AD*, n = 11/7)].

We have shown previously that leptin downregulates expression of the γ-secretase components, particularly presenilin [28]. We reasoned that leptin resistance, as seen in obesity and diabetes, would likely increase PS1 expression in the brain and, as a consequence, β-amyloid deposition in the *db/AD* mice. In order to visualize the AD pathology, brains from middle-aged mice were sectioned and stained for Aβ (Ab4G8 – specific for Aβ_17–24_). As expected, only mice containing the AD-related mutations in *APP* and *PS1* were positive for Aβ-containing plaques (Figure 2a-d). Quantitation of plaque burden indicated that the number of plaques present in *db/*AD mice decreased relative to AD mice (0.104 ± 0.012 vs. 0.168 ± 0.013 A.U.; p < 0.001; n = 9 *AD*, 10 *db/AD*; *not shown*). Contrary to the effect on plaques, the db mutation increased expression of PS1 protein (Figure 2e; p < 0.002) Expression of BACE1 (*Lepr*^*+/+*^*0.89 ± 0.06; Lepr*^*db/+*^0.87 ± 0.03*; Lepr*^*db/db*^ 0.80 ± 0.05 A.U.) and BACE2 (*Lepr*^*+/+*^*0.83 ± 0.05; Lepr*^*db/+*^0.85 ± 0.03*; Lepr*^*db/db*^ 0.84 ± 0.04 A.U.), the β-secretase proteins, was unaffected by the db genotype (n = 8 F/7 M *Lepr*^*+/+*^; 13 F/11 M *Lepr*^*db/db*^; 22 F/28 M *Lepr*^*db/+*^: p ≥ 0.41 for both proteins: *not shown*). The db mutation significantly increased tau phosphorylation (Figure 2f: p < 0.003), although no neurofibrillary tangle pathology was observed (*not shown*). On the other hand, Aβ oligomers were significantly elevated in diabetic mice (Figure 2g: p < 0.0005). Young (3 months) *AD* and *db/AD* mice had approximately equal amounts of total Aβ (Figure 2h: p < 0.3). At 6 and 12 months, there was still no overall effect of the db genotype on Aβ levels (Figure 2i-j: p < 0.23 for Aβ_total_: p < 0.07 for Aβ_1–40_) though there was a modest, but significant, reduction in Aβ_1–42_ (Figure 2k: p < 0.01).

**Figure 3:**
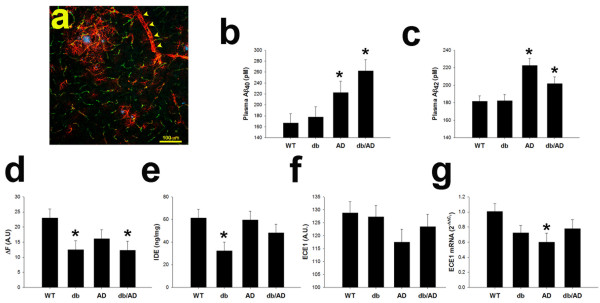
**Aβ is not deposited in the vasculature.** Large numbers of activated astrocytes could be seen both around amyloid cores and around some larger blood vessels *(arrowheads)* in older *db/AD* mice **(a)** There was no significant co-staining of amyloid and collagen IV, indicating that Aβ was not deposited in blood vessels. Red: GFAP, Green: Collagen IV, Blue: Amylo-Glo. *db/AD* mice did not have significantly more Aβ_40_ **(b)** or Aβ_42_ **(c)** in plasma, compared with *AD* mice (n = 18 F *WT*; 18 F *db*; 14 F *AD*; 13 F *db/AD*: 7–12 months old: p > 0.06), though mice containing the AD mutations had significantly more than those without (p ≤ 0.0001 for *AD* overall). Though the activities were significantly different in mice homozygous for the *db* mutation, neprilysin (**d**; p < 0.02) and insulin degrading enzyme (IDE: **e**; p < 0.003) activities were not increased relative to *db* WT mice (1–4 months old: n = 5 F/7 M *Lepr* ^*+/+*^; 8 F/4 M *Lepr* ^*db/db*^; 6 F/6 M *Lepr* ^*db/+*^). Endothelin converting enzyme (ECE1: **f)** protein expression was unaffected by the *db* (p < 0.65) and *AD* (p > 0.1) genotypes (7–12 months old: n = 14 F/7 M *WT*; 16 F/5 M *db*; 12 F/5 M *AD*; 12 F/6 M *db/AD*). Similarly ECE1 **(g)** and ECE2 (*not shown*) mRNA expression was unchanged in diabetic mice (p > 0.38 for the *db genotype* overall), though ECE1 expression was reduced in *AD* mice relative to *WT* (* = p < 0.03: n = 1 F/4 M *WT;* 2 F/4 M *db*; 1 F/3 M *AD*; 1 F/3 M *db/AD*).

Cerebral amyloid angiopathy (CAA), in which Aβ is deposited in the vasculature, is a co-morbidity in many AD patients [29]. We therefore hypothesized that excess Aβ was created, but was deposited in blood vessels rather than the brain parenchyma in the *db/AD* mice. To investigate this possibility, we pursued a variety of histopathological staining techniques. There was no appreciable Congo Red, Thioflavin S, or resorufin staining in any of the aged *db/AD* mice tested, nor did we detect any Aβ immunoreactivity in large or small blood vessels (*not shown*). Additionally, there was no co-labeling of amyloid (by Amylo-Glo) and anti-collagen IV-labeled vessels (Figure 3a). These data demonstrate a lack of vascular deposition and indicate that CAA is not a primary pathology in these mice. Additionally, we hypothesized that excess Aβ could have been produced, but cleared from the brain at an increased rate in the *db/AD* mice. We therefore measured plasma Aβ levels. Neither Aβ_1–40_ (Figure 3b: p < 0.18) nor Aβ_1–42_ (Figure 3c: p ≤ 0.06) were significantly increased in the plasma of older *db/AD* mice, compared to *AD* mice. We also reasoned that increased activity of clearance enzymes could also account for a loss of Aβ. However, neither NEP (Figure 3d) nor IDE (Figure 3e) activities were increased by the *db* genotype. In fact, the diabetic mice displayed significantly reduced activities of these major clearance enzymes (NEP; IDE; p ≤ 0.01 for both), compared to non-diabetic mice. Expression of endothelin-converting enzyme (ECE1) protein was unaffected by the *db* genotype (Figure 3f: p < 0.65). Similarly, mRNA expression of ECE1 (Figure 3g) and ECE2 (*not shown*) was unaffected by genotype (n = 1 F/4 M WT; 2 F/4 M db; 1 F/3 M AD; 1 F/3 M *db/AD*: p ≥ 0.13 for both genes).

### Cognitive deficit

**Figure 4:**
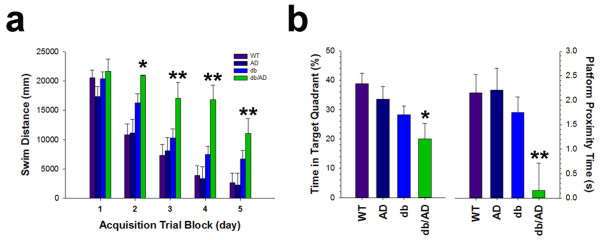
***db/AD*** **mice display impaired cognition.** *db/AD* mice showed a significant acquisition deficit in the Morris Water Maze **(a)**: ANOVA p-value by day: 0.4, 02, 0.05, 0.001, 0.03) using a standard paradigm of 4 trials/day (mean swim distance is shown for each block: (*WT*, n = 4 F/4 M; *AD*, n = 4 F/2 M; *db*, n = 6 F/5 M; *db/AD*, n = 7 F/1 M). **(b)** *db/AD* mice did not learn the location of the hidden platform, as shown by probe trial (ANOVA, p < 0.03). The *db/AD* mice spent less time in the target quadrant, and less time in proximity to the platform location (* = p ≤ 0.05, ** = p ≤ 0.01. Tukey’s test, relative to *WT*). All groups performed similarly on the cued version of the task (both distance (p < 0.07) and latency (p < 0.12), indicating that the *db* and *db/AD* mice did not have a profound visual impairment (*not shown*).

Since the diabetes phenotype did not significantly impact amyloid accumulation, we next determined if there was an effect on cognition in these mice. We tested older mice using a standard Morris Water Maze paradigm. While there was no difference in swim distance on the first day of training (Figure 4a: p < 0.38), the *db/AD* mice had significantly longer swim paths on each of the subsequent days (p ≤ 0.04 on each of days 2–5), indicating a learning and/or memory impairment. Neither *db* nor *AD* mice showed any significant impairment at this age (p ≥ 0.13 for each genotype). Swim speeds were not significantly different on the first training day (*WT* 374 ± 18; *AD* 356 ± 21; *db* 368 ± 18; *db/AD* 352 ± 18 mm/s: p ≥ 0.4 for all comparisons: *not shown*) demonstrating that *db/AD* mice are capable of swimming as well as other genotypes. After the fifth day of acquisition trials, we performed a probe trial during which the platform was removed. In this trial, *db/AD* mice spent significantly less time in the target quadrant (Figure 4b: p < 0.04) and less time in proximity to the platform location (p < 0.01). Since diabetics are prone to retinopathy and blindness, we wanted to exclude a profound visual impairment that would affect performance on the Morris Water Maze. We, therefore, performed a visual acuity test in which the platform location was visible and cued. All animals, with the exception of one *db/AD* mouse, were able to locate the cued platform (χ^2^ = 3.22: p < 0.36). We also analyzed the visual acuity data using an ANOVA: the *db* genotype had no effect on either swim distance (*WT* 2610 ± 2059; *AD* 3576 ± 2511; *db* 5805 ± 1757; *db/AD* 9700 ± 3173 mm: p < 0.07: *not shown*) or latency to platform (*WT* 10.6 ± 3.4; *AD* 11.7 ± 3.6; *db* 13.45 ± 2.4; *db/AD* 21.6 ± 4.5 seconds: p < 0.12: *not shown*), indicating that visual impairment could not account for the deficit. These data indicate that intersection of the both diabetes and AD is necessary for cognitive impairment.

### Synapse loss

**Figure 5:**
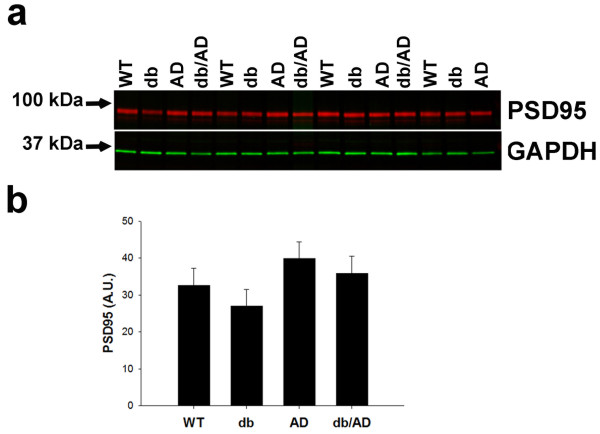
**Synapse Loss in** ***db/AD*** **mice. (a)** A representative immunoblot of PSD95 expression in brains from older *db/AD* mice. The immunoblot was visualized with an Odyssey Infrared Imager (LI-COR). Red = PSD95, Green = GAPDH. **(b)** Analysis of PSD95 expression in the four main genotypes. PSD95 expression was standardized to that of GAPDH in the same lane. PSD95 expression was unaffected by either the *db* (p > 0.3) or the *AD* (p > 0.09) genotype.

Because synaptic dysfunction and subsequent loss have been implicated in AD- associated memory impairment [2–4], we next measured the amount of the synaptic marker PSD95 in older mice. Neither diabetes nor the *AD* genotype significantly affected the level of PSD95 in the brain (p > 0.1: Figure 5), indicating that the number of synapses is not substantially reduced in the *db/AD* mice at the age at which we have observed learning and/or memory deficiencies.

### Cerebrovascular abnormalities

**Figure 6:**
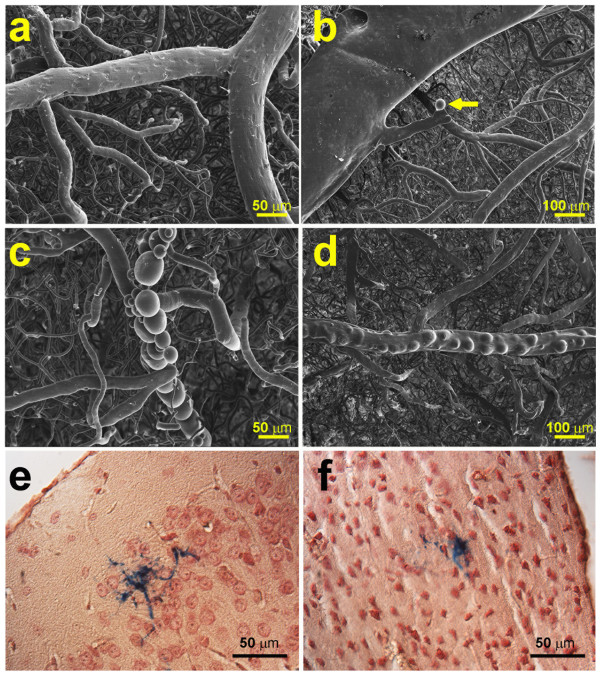
**Cerebrovascular pathology in** ***db/AD*** **mice.** SEM images of vascular casts from brains of 4 mice **(a-d)**. **(a)** Relatively normal appearing cerebrovasculature of a *WT* mouse (large vessel is a small artery; note the clear endothelial cell nuclear imprints and their elongated shapes). **(b)** Aneurysm (*arrow*) in the brain of an *AD* mouse near a large vein. Some of the *AD* mice exhibited more severe cerebrovascular pathology, possibly representing clusters of saccular aneurysms **(c)** or arterial blebbing **(d)**. In comparison, *WT* and *db* mice had minimal pathology at this age. **(e - f)** Prussian blue (with neutral red counterstain) staining showing microhemorrhages in two different cortical areas in older *db/AD* mice.

Since the learning and memory deficit in *db/AD* mice was not obviously attributable to accumulation of Aβ or synapse loss, we next focused on changes in the cerebrovasculature. We examined the brain vasculature in older mice using vascular corrosion casting followed by scanning electron microscopy. *WT* and *db* mice had normal appearing vasculature (Figure 6a). By contrast, *AD* and *db/AD* mice displayed a marked pattern of cerebrovascular pathologies. We observed evidence of widespread saccular aneurysms, often occurring at the vessel branch points (Figure 6b). Some of the mice tested also presented with extensive clusters of apparent aneurysms along the arteries and arterioles (Figure 6c) as well as arterial blebbing that may represent weakened areas of the vessel wall (Figure 6d). We next scored the aneurysm pathology on a four-point scale (0 = none; 1 = 1 possible; 2 = 1–3 definite; 3 = 4+ definite) and found that aneurysms were significantly more numerous in mice with the *AD* genotype (*AD and db/AD* mice 1.7 ± 0.23 vs. *WT* and *db* mice 0.45 ± 0.24: F[1, 15] = 14.14, p < 0.002 for the *AD* genotype overall; N = 4-5 F/ genotype: *not shown*). The presence of the diabetes genotype did not significantly increase the number of aneurysms (p < 0.2 for the *db* genotype overall).

Because aneurysms are unstable and prone to rupture, we next looked for evidence of hemorrhage in the *AD* and *db/AD* mice using Prussian blue staining for hemosiderin. Prussian blue staining showed a significant incidence of microhemorrhages in older *db/AD* mice (n = 7; χ^2^ = 4.75, p < 0.03); we did not find microhemorrhages in genotypes other than the *db/AD* (n = 9: Figure 6e-f), including *AD* mice, which also displayed significant aneurysm pathology.

**Figure 7:**
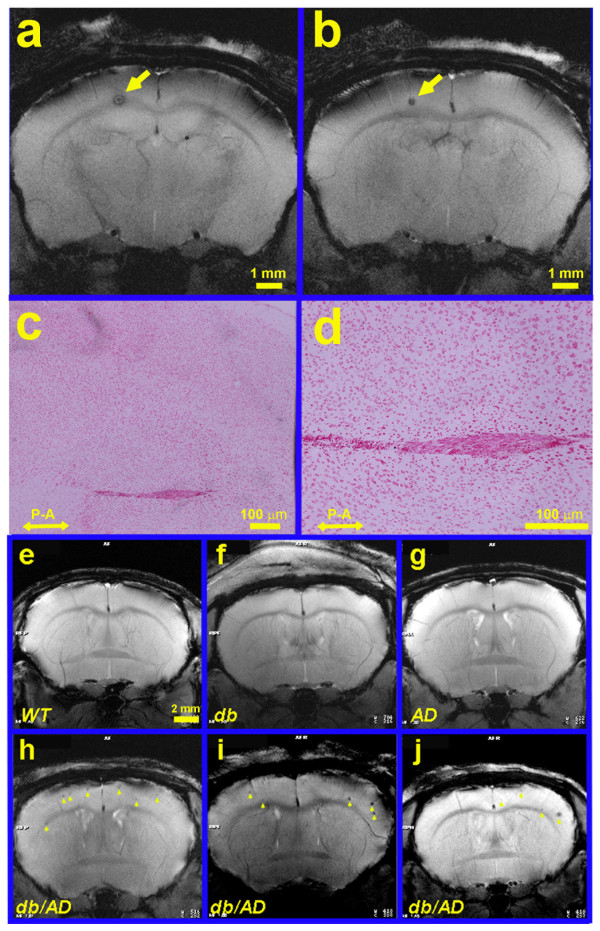
**Stroke pathology in** ***db/AD*** **mice.** Sequential (**a** posterior to **b**) T2*-MRI coronal images from an older *db/AD* mouse showing a neocortical event (*arrow*) near the corpus callosum; images are separated by ~300 μm. **(c - d)** The brain was sectioned transversely to obtain confirmation of stroke extent. Prussian blue staining with neutral red counterstain showed no evidence of hemorrhage, indicating an ischemic stroke event. **P – A**: posterior / anterior, for orientation (*N.B.:* section is perpendicular to scanning axis). **(e- j)** ~70% of *db/AD* mice (n = 11/15) had strokes *(arrowheads);* these were rare in *AD* mice (**g**: n = 2/9), and not found at all in *WT* (**e**: n = 9) or *db* mice (**f**: n = 10). All mice imaged were 12–14 months old. The *db/AD* mice **(h - j)** often have multiple incidents as opposed to the two *AD* mice, which only displayed one or two small strokes. Representative cases are shown (all scans are at about the same neuroanatomical level).

We scanned a separate cohort of older mice using small animal magnetic resonance imaging (MRI) in order to visualize areas of hemorrhage and infarcts. Indeed, the majority of the *db/AD* mice tested (11/15) showed evidence of multiple vascular events by MRI (Figure 7a-b, h-j). Histological staining of brains from the scanned mice was negative for Prussian blue staining (indicating the lack of hemorrhage). Moreover, micrographs from the same neuroanatomical level as the largest event detected by MRI showed obvious necrosis in the surrounding tissue and an obvious lack of Prussian blue positive staining (Figure 7c-d). The histological data suggest that the vascular events are likely ischemic strokes. By contrast, no *WT* (0/9: Figure 7e) or *db* (0/10: Figure 7f) and only a small number of *AD* (2/9: Figure 7g) mice presented with strokes, suggesting that the presence of both the *db* and *AD* genotypes is required to promote these events (χ^2^ = 21.769; p ≤ 0.0001). In addition, there were multiple events present in the *db/AD* mice (Figure 7h-j), whereas only one or two were present in the *AD* mice positive for strokes.

### Passive immunization

Our data suggest that the intersection of the *db* and *AD* genotypes is necessary to induce strokes in these mice. In light of this data and the absence of diabetes-induced amyloid accumulation, we believe that the stroke pathology is unlikely to be due to Aβ accumulation. In order to test this hypothesis, we next performed a pilot study in which we immunized older *db/AD* mice with an Aβ antibody (Ab42.5) for two months. Parenchymal Aβ was significantly reduced via immunization compared to age-matched, untreated db/AD mice (~17% decrease; n = 6 / group; p < 0.006: *not shown*). Though Aβ was significantly reduced in the brain, there was no evidence that the stroke phenotype was rescued. Most of the treated mice imaged by MRI showed evidence of stroke (4/6 treated vs. 11/15 untreated; p < 0.67: *not shown*).

### Vascular density

**Figure 8:**
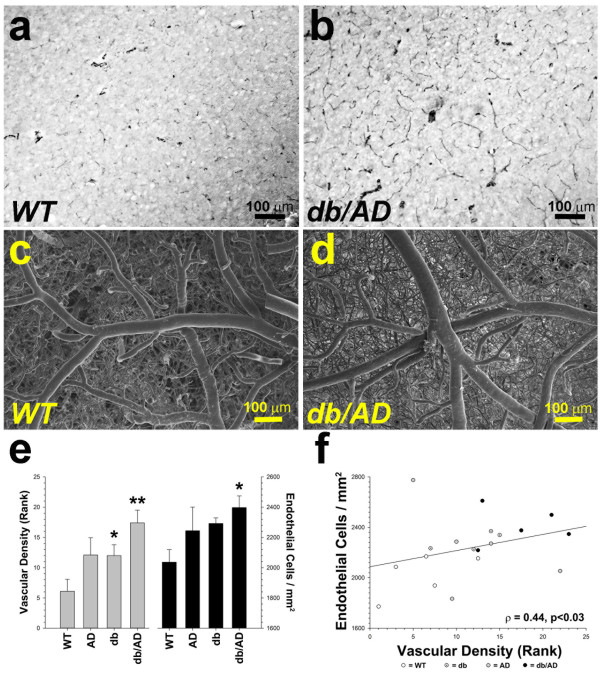
**Vascular and endothelial density increase in** ***db/AD*** **mice. (a - b)** Immunohistochemical staining for smooth muscle α-actin indicates that the brains of *db/AD* mice are more vascularized. **(c - d)** SEM images of the brains subjected to vascular corrosion casting show a similar increase in the density of the cerebrovasculature. **(e)** Median vascular density scores of SEM images from three blinded, independent raters (* = p ≤ 0.05, ** = p ≤ 0.01 compared to *WT*, Mann–Whitney U-test). Endothelial cells were also directly counted on five randomly-selected arteries / animal (* = p ≤ 0.05 compared to *WT*; Kruskal-Wallis ANOVA). Direct measurement of cell size (68 ± 27 cells / animal) from the middle cerebral artery indicated that *db/AD* endothelial cells were smaller (F[3,8] = 7.8, p < 0.01) than those from *WT* mice, and as expected size was inversely correlated with density (*R* ^*2*^ = 0.41, p < 0.02; *not shown*). **(f)** Endothelial cell density was also correlated with vascular density (p = 0.03).

γ-Secretase has been implicated in the regulation of VEGF-dependent angiogenesis [30–32]. Since we observed an upregulation of PS1 expression in the *db/AD* mice in the absence of Aβ accumulation, we hypothesized that PS1 might contribute to the observed vascular pathology through the regulation of angiogenesis. We therefore measured the amount of blood vessels present in the brains of older *db/AD* mice. Staining for smooth muscle α-actin indicated that the brains of *db/AD* mice were significantly more vascularized than those of *WT* mice (1.35 ± 0.52 vs. 0.31 ± 0.52: n = 4/ genotype: p < 0.04: Figure 8a-b). SEM images showed a similar increase in the density of the cerebrovasculature in the brains subjected to vascular corrosion casting (Figure 8c-d). Indeed, median vascular density scores of the SEM images from three blinded, independent raters indicated that the *db* genotype significantly increased vascular density (Figure 8e: N = 19: p < 0.02, Mann–Whitney U-test). The *AD* genotype had a marginal effect, (p ≤ 0.05). Similarly, *db/AD* mice had a greater number of endothelial cells than the other genotypes (p ≤ 0.05; Kruskal-Wallis ANOVA), as measured by the endothelial cell nuclear imprints. Endothelial cell density was correlated with vascular density (Figure 8f: p < 0.03). Direct measurement of cell size (68 ± 27 cells / animal) from the middle cerebral artery indicated that endothelial cells were smaller in diabetic mice (F[3, 8] = 17.9, p < 0.01), and size was inversely correlated with density (*R*^*2*^ = 0.41, p < 0.02). Collectively, these data indicate that *db/AD* mice had an increase in the number of cerebral blood vessels, supportive of increased angiogenesis or arteriogenesis.

## Discussion

### The db/AD model

We have created a unique mouse model that encapsulates features of both T2DM and AD- the *db/AD* mouse. These mice are morbidly obese and glucose intolerant at a young age (Figure 1a-d), and have a profound cognitive impairment by 12 months (Figure 4). The *db/AD* mice display decreased survival (Figure 1e-f), the cause of which is currently unknown. Male *db/AD* mice appear to be more susceptible to premature death, though sexual dimorphism has been noted in many AD models [33–35]. While their lifespan is shortened relative to control genotypes, we were able to routinely age the *db/AD* mice beyond 12 months, allowing significant Aβ accumulation, plaque formation, stroke pathology, and cognitive impairment.

Contrary to our expectations, the *db/AD* mice did not exhibit increased parenchymal Aβ accumulation compared with the normoglycemic *AD* mice (Figure 2h-k), in spite of the observed increase in PS1 expression (Figure 2e). Aβ oligomers were modestly elevated in both *db* and *db/AD* mice (Figure 2g), though the potential impact of this increase is unknown at this point. It is possible that the detected oligomers are formed from murine Aβ and, thus, are not toxic. The reason for the relative dearth of excess Aβ in *db/AD* mice is unclear, though it does not appear to be due to stimulation of clearance mechanisms. While we cannot rule out clearance by other enzymes, the major enzyme activities that proteolyze Aβ (neprilysin, IDE, and ECE) were not increased in *db/AD* mice (Figure 3d-g), nor was there an increase in peripheral Aβ in the plasma (Figure 3b-c). In addition, we found no evidence that Aβ is deposited in the vasculature (Figure 3a), despite using multiple different staining techniques. Based on this data, it is likely that excess Aβ is simply not made in *db/AD* mice. In addition, there is no evidence of a significant reduction in the number of synapses in older *db/AD* mice (Figure 5). These findings indicate that neither CAA nor synaptic loss causes the cognitive decline observed in our mouse model.

The most striking feature of this mouse model is the severe vascular abnormalities that are present, apparently in the absence of a corresponding increase in Aβ deposition. Older *AD* and *db/AD* mice exhibited profound aneurysm pathology (Figure 6b-d) and *db/AD* mice had small strokes (Figure 7). Though we did observe a few areas of hemosiderin-positive staining in those animals with the largest number of vascular events (Figure 6e-f), we did not see substantial numbers of hemorrhages in the *db/AD* animals. Indeed, it is possible that the more extensive pathologies observed by SEM are representative of ischemic stroke, but take on this appearance during the vascular corrosion casting process. In addition, the largest event observed by MRI (Figure 7a-b) did not stain positive for hemosiderin (Figure 7c-d) and was likely ischemic in nature. We feel that infarction is the likely cause of these events, but further characterization will be needed. This is broadly consistent with the type of cerebrovascular disease observed in human diabetics [36, 37]. Given that the *db/AD* mice were the only genotype to exhibit both stroke pathology and cognitive impairment, we believe that it is these strokes that are responsible for the observed cognitive decline.

### Mechanism of vascular pathology

Based on our data, it is likely that the aneurysm and stroke pathologies are separable events. Aneurysms were prevalent in *AD* animals, regardless of diabetic phenotype and were not exacerbated by diabetes. This suggests that the aneurysms may be caused by some feature of the *AD* genotype. While aneurysms are not typically associated with AD in humans, increased blood vessel tortuosity, which is associated with aneurysms in other diseases, has been observed [38, 39]. In addition, mutations in the presenilin substrate Notch are associated with thoracic aneurysms, likely through crosstalk with TGFβ signaling [40, 41]. The mutation in PS1 present in the *AD* mice may also affect this Notch signaling pathway, resulting in the aneurysm pathology.

On the other hand, the intersection of the *db* and *AD* genotypes was necessary to induce strokes in these mice (Figure 7). In light of this data and the absence of diabetes-induced amyloid accumulation, we believe that the stroke pathology is unlikely to be due to Aβ accumulation. This hypothesis was supported by preliminary data from our passive immunization study, which showed that stroke incidence was not reduced in *db/AD* mice treated with an anti-Aβ antibody, though brain Aβ levels did decrease. While interesting, a more extensive study will be needed for a more definitive conclusion.

Diabetes itself has profound effects on the vasculature. Obesity and diabetes are associated with hypertension and atherosclerosis [42]. In addition, diabetic rodents, including *db/db* mice, have increased neovascularization such as angiogenesis and arteriogenesis [43–45]. This neovascularization consists of immature, unstable blood vessels that display increased permeability of the blood–brain barrier. Similar pathologic angiogenesis occurs in diabetic retinopathy and is thought to involve presenilin and γ-secretase regulation of VEGF signaling [30, 46]. We have evidence that PS1 expression increased in diabetic mice (Figure 2e) regardless of the AD mutations present- as expected with the use of “knocked-in” genes under endogenous promoters. Consistent with PS1 upregulation, the *db/AD* mice have a significantly higher density of blood vessels in the brain than any of the other genotypes tested (Figure 8). Further studies will be needed to determine if neovascularization may indeed play a role in the strokes and/or cognitive impairment, or if some other diabetes-related phenomenon underlies these pathologies. We have shown previously that leptin downregulates PS1 expression in both *in vitro* and *in vivo* models [28]. It will be interesting to determine if leptin resistance in the *db/AD* mice contributes to neovascularization via regulation of the γ-secretase complex.

### A unique model of mixed dementia

The form of dementia afflicting diabetic individuals combines elements of vascular pathology, small strokes and AD-related neuropathology. In fact, the amount of AD pathology is essentially unchanged in cases with a history of T2DM, while cerebrovascular pathology increases [9, 10]. The *db/AD* mice share these features. One way of looking at this seemingly paradoxical observation is that cerebrovascular pathology lowers the threshold for incipient AD pathology to become unmasked as a clinical dementia as has been suggested elsewhere [10].

A small number of studies have examined the linkage between obesity, diabetes and dementia in rodent models [47, 48]. The majority of these are focused on two paradigms: treatment with streptozotocin (STZ) and feeding a high fat, or typical Western, diet (TWD). STZ, a pancreatic islet toxin, is primarily used to model type I diabetes; thus it does not address the issue of obesity. Although TWD feeding induces obesity, and has some short-term effects on AD-related neuropathology in these models [49–51], these studies have failed to provide any detailed mechanistic insights into how obesity might influence the development of age-related neurologic disease. Further, TWD feeding does not have strong long-term effects on AD and vascular dementia-related neuropathology [52]. Studies utilizing genetic models of diabetes have been more limited. When *Tg2576* mice, which overexpress *APP*^*ΔNL*^, are crossed with *Irs2*^−/−^ insulin resistant mice, the resulting animals show reduced amyloid pathology [53]. In addition, a recent study examined the outcome of a cross between leptin-resistant *ob/ob* mice and *APP23* mice [54]. These animals showed a very early Morris Water Maze deficit (2–3 months old) unrelated to amyloid load, as the animals had no plaques and no differences in Aβ levels compared with non-diabetic controls. Even at the oldest age examined (12 months old), plaque pathology in these mice was virtually nonexistent, although there was some vascular amyloid in a very small number of animals (n = 3). The choice of parental mouse lines has a profound effect on the viability of the resulting mice as well: a cross between the *ob/*ob and *Tg2576* lines yielded animals with significantly reduced viability [55]. While our data are in broadly supportive of these other studies, the *db/AD* mice are unique in that they have Aβ plaques, very little vascular-associated Aβ, and profound underlying vascular abnormalities, even in the absence of a high-fat diet.

In summary, the *db/AD* mouse is a unique model of mixed dementia, possessing both AD-related and vascular pathologies. Older mice present with extensive stroke pathology, arising from a combination of the diabetic and AD phenotypes, thus leading to significant cognitive impairment. While these data suggest that Aβ is not a primary factor in the observed cognitive impairment, we cannot exclude the possibility that a soluble form of Aβ, such as oligomers, may play a role in the cognitive decline. Future studies will focus on the mechanisms behind the vascular abnormalities, at both the cellular and tissue levels. Finally, the *db/AD* mouse is a novel model in which to test possible therapeutic and preventative strategies to treat cognitive decline from mixed dementia.
